# The association of different types of human milk with bronchopulmonary dysplasia in preterm infants

**DOI:** 10.3389/fnut.2024.1408033

**Published:** 2024-08-07

**Authors:** Elisabeth Pütz, Rudolf Ascherl, Thomas Wendt, Ulrich H. Thome, Corinna Gebauer, Jon Genuneit, Linda P. Siziba

**Affiliations:** ^1^Pediatric Epidemiology, Department of Pediatrics, Medical Faculty, Leipzig University, Leipzig, Germany; ^2^Division of Neonatology, Department of Pediatrics, University of Leipzig Medical Center, Leipzig, Germany; ^3^Data Integration Center, University of Leipzig Medical Center, Leipzig, Germany; ^4^German Center for Child and Youth Health (DZKJ), Leipzig, Germany

**Keywords:** bronchopulmonary dysplasia (BPD), donor human milk (DHM), human milk, Leipzig Donor Human Milk Bank (LMB), pasteurization, mother’s own milk (MOM)

## Abstract

**Objective:**

To evaluate the association between different types of human milk feeds and bronchopulmonary dysplasia (BPD) in preterm infants.

**Methods:**

Data on dispensed mother’s own milk (MOM) and donor human milk (DHM) from Leipzig Milk Bank for hospitalized infants with a gestational age (GA) ≤32 weeks observed from birth to 36 weeks’ postmenstrual age or prior discharge were used. BPD was assessed based on documented International Statistical Classification of Diseases and Related Health Problems (ICD) diagnosis and on electronic hospital records (EHR) of data on ventilation and oxygen supplementation. Associations of dispensed milk feed variations with BPD were investigated using logistic regressions in crude and adjusted models.

**Results:**

866 infants were included with a BPD prevalence of 15.4% (EHR) and 23.2% (ICD). The mean GA was 29.1 weeks. The majority (84.4%, *n* = 746) of infants were nurtured with a mix of MOM, DHM supplemented by formula or parenteral (other) nutrition during hospitalization. For which, MOM comprised the highest median [Q1–Q3] percentage proportion (53[31–81] %) of this mix. Exclusive fresh milk and exclusive MOM feeds were dispensed on a mean of 40 and 34% patient-days, respectively. Statistically significant associations with lower BPD incidence were only observed for 70–80% MOM vs. DHM, and 60% fresh vs. frozen milk, in crude and adjusted models.

**Conclusion:**

Our findings suggest a protective association of MOM and fresh milk with lower odds of BPD, which may be dependent on the proportion of MOM or fresh milk administered. These results highlight the importance of MOM as an ideal source of nutrition during early infancy.

## 1 Introduction

Human milk is the gold standard of enteral nutrition for preterm infants, promoting their health. When mother’s milk is not enough or available, donor human milk (DHM) is preferred over preterm formula (PF) (1, 2). The beneficial effects of human milk among very low birth weight (VLBW) infants, and particularly in reducing risks of necrotizing enterocolitis (NEC), late-onset-sepsis (LOS), and bronchopulmonary dysplasia (BPD) are well documented (3–8). These benefits are attributed to its high content of bioactive components, antioxidant agents and immune modulating factor that support the infant’s immune system (9, 10).

BPD, a chronic lung disease and common complication after preterm birth, especially affects infants born at <28 weeks’ gestational age (GA), with higher risk of mortality, morbidity, and long-term pulmonary consequences (11, 12). In addition to the required parenteral nutrition for preterm infants, optimal and early enteral nutrition is considered to be essential for normal lung development and might have a protective effect against BPD (13–15). However, it is uncertain whether a specific type of human milk (e.g., MOM or DHM) has an influence on the development of BPD.

Studies have shown that the macronutrient and micronutrient composition of MOM and DHM differs (16, 17), which might have adverse infant outcomes such as BPD. However, comparisons between MOM and DHM feeds do not show MOM’s superiority against BPD (18, 19). Studies comparing raw (unpasteurized) human milk with pasteurized human milk are scarce and findings are inconclusive (20, 21). Pasteurization may compromise human milk’s immunological components, increasing the risk of infection-related complications, including BPD (22, 23). Additionally, freezing and thawing human milk may have similar effects (24). Overall, evidence is limited, with few studies comparing different human milk types and their associations with infant outcomes, as noted in our previous scoping review (25).

A limitation of previous studies is that the assignment into comparison groups by percentage or patterns of human milk fed is heterogeneous and making it challenging to interpret and compare results (26). Furthermore, the relative proportions of MOM and DHM to categorize infants by their nutrition are usually not reported in these studies (17). The use of arbitrary cut-offs (e.g., 75% DHM) may not be applicable in otherwise different populations and settings (27).

The definition of BPD has been revised several times over the past years, based on several additional parameters (28). Of note, there is currently no worldwide accepted or practiced BPD definition (29). Thus, we used two distinct operationalizations of BPD in this study: (i) a main or secondary diagnosis of BPD using the International Statistical Classification of Diseases and Related Health Problems (ICD-10-GM, code P27.1) in administrative hospital data and (ii) classification of BPD cases based electronic hospital record (EHR) data on ventilation and oxygen needs following the National Institute of Child Health & Human Development (NICHD) consensus definition from 2000 (30), similar to previous studies (3, 19, 31). Therefore, this study’s objective was to investigate the associations of fresh, frozen, raw and pasteurized MOM and/or DHM milk feeds dispensed from the Leipzig Donor Human Milk Bank (LMB) with BPD in infants born at ≤32 weeks GA, using different cutoffs and the two afore-mentioned operationalizations of BPD.

## 2 Methods

### 2.1 Data sources and study variables

The LMB of Leipzig University Medical Center provides a supply of human milk for infants whose mothers cannot express human milk. Retrospective data on daily dispensed milk feeds from 2012 up to 2019 comprising MOM, DHM, fresh, frozen, raw and pasteurized milk were used for the current analysis (32). These data were merged with electronic hospital records by the Data Integration Center (Datenintegrationszentrum; DIZ), which contained additional information on general characteristics like child sex, age at admission, birth weight, morbidity, and parameters required to derive the BPD outcome. All data were restricted to infants that were born at ≤32 weeks GA. The observational period for infants <32 weeks GA was from birth to 36 weeks’ postmenstrual age or discharge, whichever occurred first, i.e., the time at which BPD diagnosis is established. For children with exactly 32 weeks GA, per NICHD consensus definition, the observational period was >28 but <56 days’ postnatal life or discharge. The Ethics board of the Medical Faculty at Leipzig University approved this study (#026/23).

In principle, frozen milk is stored for a maximum of 6 months at −20°C and chilled milk is kept for a maximum of 3 days at 4°C. DHM is pasteurized if the donor is cytomegalovirus (CMV) positive or the skin microbial count is over 10^4^/mL, whereas MOM is pasteurized for preterm infants with a GA of <28 weeks and CMV positive mothers until 32 weeks GA. For MOM, a maximum skin microbial count of 10^5^ is acceptable. The milk is processed by Holder Pasteurization (62.5 degrees for 30 min) (32). In addition, the milk is fortified with a bovine based multi-component fortifier for preterm infants, mostly with a birth weight < 1,500 g, when an enteral intake of 70–100 mL/kg/day milk is reached. However, there was no information on actual milk volume, actual milk fed to the infant and/or biological composition of MOM or DHM. Thus, data on dispensed human milk feeds were weighted by the inverse of the count of different milk types dispensed on a given day as a proxy for the changing feeding type. The numerical contributions of these milk types were then subsequently determined. Although these data may be prone to some error, for instance, if clinically required nutrition requirements changed during the day, we previously showed plausible complex intra-individual patterns during the first 100 days of hospitalization (32). These findings highlighted the importance of selecting cut-offs based on the underlying research population and the desired contrast. Thereby potentially improving accuracy in grouping infants into distinct human milk feeds accounting for co-exposure to each milk feed.

### 2.2 BPD definition

**Figure 1:**
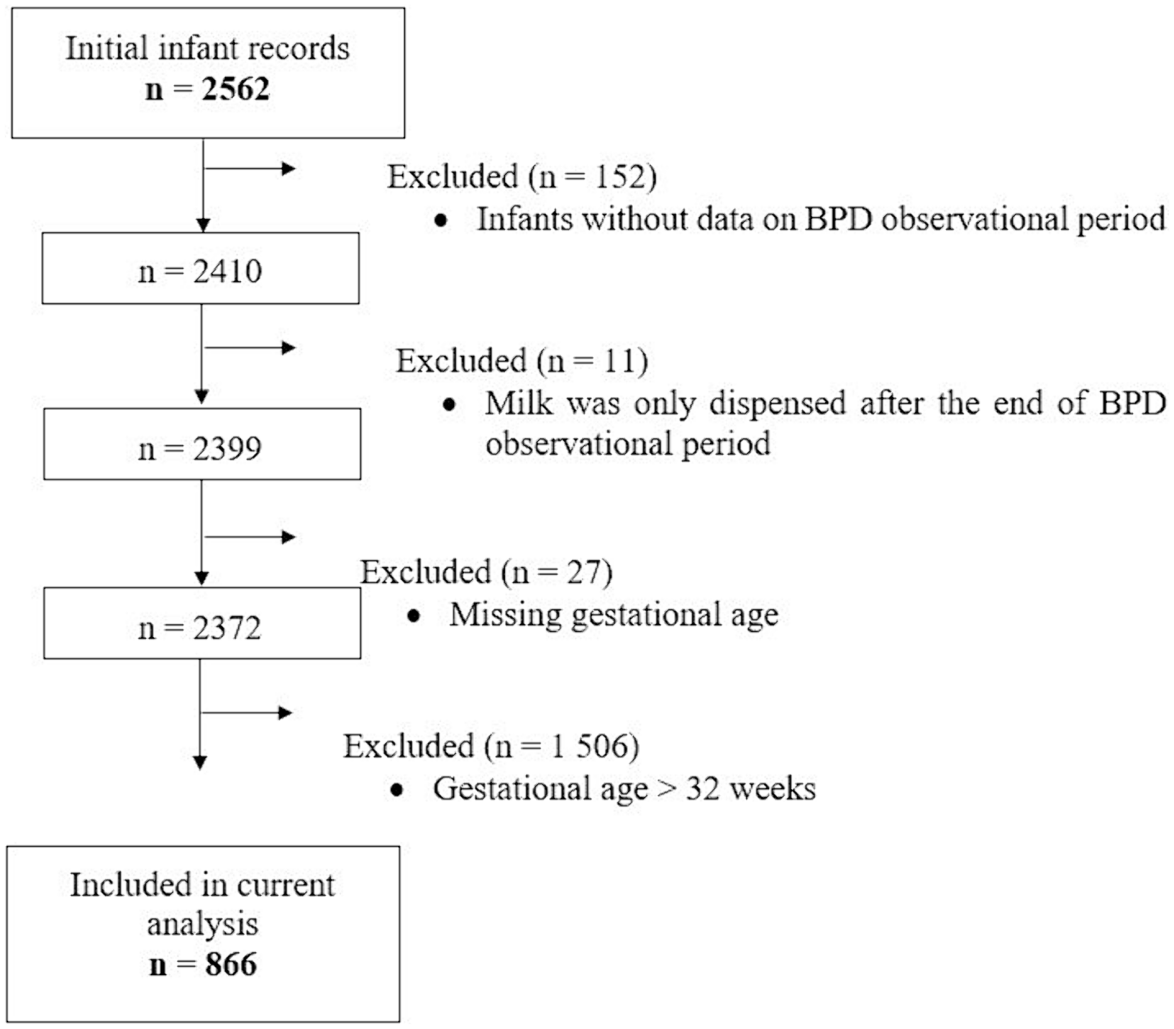
Flowchart of hospitalized infants whose records on dispensed milk types were used for the current analysis. BPD, Bronchopulmonary Dysplasia.

The first BPD operationalization was the extraction of a documented physician’s diagnosis based on the ICD-10-GM, code P27.1 from the electronic discharge summary used for administrative purposes. For the second operationalization, the ventilation parameters and oxygen supply over the course of hospitalization were extracted from the patients’ electronic hospital records (EHR). These included high-resolution data from medical ventilation devices and patient monitors. The data were subjected to an automated algorithm to identify BPD cases according to the consensus definition formed by the NICHD Workshop in 2000 (30). Specifically, treatment with oxygen >21% for at least 28 days meant that the infant received oxygen >21% for more than 12 h on each of the 28 days, which had to be achieved cumulatively but not necessarily consecutively in the observational period (see above) from birth. BPD severity based on EHR data was investigated, although sample size was very low. However, BPD severity was not reflected in the other operationalization by ICD code. Data on dispensed milk feeds were available from a total of 2,562 infants, but only 866 infants born at ≤32 gestational weeks had complete data set of dispensed milk, BPD outcomes, and other relevant characteristics for analysis (Figure 1).

### 2.3 Statistical analysis

Data on daily dispensed milk feeds were analyzed during the observational period according to the milk type, classified by MOM/DHM, fresh/frozen and pasteurized/raw (non-pasteurized). On some patient-days, mixed milk types (e.g., a portion MOM and a portion DHM) were dispensed which was accounted for by applying weights (e.g., 0.5 in case of two components). On some patient-days, no human milk was dispensed because infants received parenteral or other forms of enteral nutrition; these patient-days were coded as days with other nutrition. Numerical contributions of each of the dispensed human milk types to the overall nutrition during the observational period were calculated as percentages of the patient-days on which the respective human milk type was dispensed. These percentages were used to determine relevant cut-offs for subsequent analyses (e.g., 50% MOM vs. 50% DHM). Kernel density plots were used to visualize the distribution of the percentages of the different milk types, analogous to a histogram. Infants were categorized into one of five groups, the first four were based on cut-offs for the distributions of the different milk types that ranged from 20 to 80%; and the fifth group was based on fresh MOM vs. any DHM in the first 14 days. Associations between dispensed milk feeds and BPD were assessed using logistic regression in crude and adjusted models. Gestational age, birth weight, child sex, surfactant therapy, and pre- and postnatal steroid therapy were identified as factors that have an influence on the development of BPD (12, 28, 30, 31) and were entered into the models as confounding factors. Gestational age is highly associated with the risk of BPD (12). Therefore, infants were stratified into two groups (GA <28 weeks and GA between 28 and 32 weeks) for subsequent analysis. All statistical analyses were done using R (version 3.5.1; R Foundation for Statistical Computing) and SAS version 9.4 (The SAS Institute, Cary, NC, United States).

## 3 Results

Baseline characteristics of the infants (*n* = 866) are presented in Table 1. Of these, 201 (23.2%) had BPD according to the ICD-based operationalization and 133 (15.4%) had BPD according to the EHR-based operationalization; *n* = 123 had both. The disparity between the methods is due to the differences in the local practice to judge a need for positive airway pressure ventilation with ambient air equivalent as a need for oxygen >21%.

**Table 1:** Characteristics of infants included in the current analysis.

	Category	Frequency (*n*)	% or mean
Infant sex	Boys	466	53.8%
	Girls	400	46.2%
Birth weight	ELBW(<1,000 g)	270	31.2%
	VLBW (≥1,000 and < 1,500 g)	286	33.0%
	LBW (≥1,500 and < 2,500 g)	308	35.6%
	Other (≥2,500 g)	2	0.2%
Gestational age (weeks)		866	29.1
Gestational age	Less than 28 weeks	226	26.1%
	28–32 weeks	640	73.9%
Milk type^1^	MOM and DHM only	15	1.7%
	DHM + other nutrition	65	7.5%
	MOM + other nutrition	55	6.4%
	MOM + DHM + other nutrition	746	84.4%
Proportions of MOM and DHM^2^	MOM and DHM only	83% [62–90%]	17% [10–38%]
	DHM + other nutrition	.	53% [21–89%]
	MOM + other nutrition	47% [32–74%]	.
	MOM + DHM + other nutrition	53% [31–81%]	16% [8–36%]
BPD: ICD operationalization	Yes	201	23.2%
	No	665	76.8%
BPD: EHR operationalization	Yes	133	15.4%
	No	733	84.6%
BPD: both ICD and EHR	Yes	123	14.2%
Antenatal corticosteroids	None	87	10.0%
	Betamethason/Dexamethason	672	77.6%
	Other	75	8.7%
	Unknown	32	3.7%
Multiples	1	586	67.7%
	2	232	26.8%
	3 or greater	48	5.5%
Surfactant application	None	192	22.2%
	LISA	114	13.2%
	Tube	545	62.9%
	LISA + Tube	15	1.7%
Sepsis	Yes	114	13.2%
	No	752	86.8%
Systemic and inhaled steroid therapy (postnatal)	Yes	204	23.6%
	No	662	76.4%

Surfactant variable includes initial care after birth and application during the further hospital stay. ^1^Refers to the children who received the respective milk type during the period of admission until end of BPD period. ^2^The median and [Q1–Q3] percentage of days, which were covered by either MOM or DHM, respectively, in each of the four categories shown. Other nutrition refers to other forms of parental and/or enteral nutrition that could have been ordered for the infants. BPD, Bronchopulmonary dysplasia; ELBW, Extremely low birth weight; LBW, Low birth weight; VLBW, Very low birth weight; GA, Gestational age; ICD, International Statistical Classification of Diseases and Related Health Problems; EHR, Electronic hospital records; LISA, Less Invasive Surfactant Administration, MOM, Mothers’ own milk; and DHM, Donor human milk.

The mean GA was 29.1 weeks; with 26.1% (*n* = 226) born before 28 weeks. The majority of infants (84.4%, *n* = 746) had a mix of MOM, DHM, and other nutrition dispensed for them, with MOM comprising the highest median percentage proportion (53%, Q1–Q3, 31–81%); i.e., 50% of the infants had MOM covering at least 53% of their hospitalized days, while for 25% of infants, MOM covered at least 81% of their hospitalized days. Dispensing exclusive MOM and DHM feeds was rare, observed in only 15 (1.7%) infants during hospitalization until the end of the BPD period.

Table 2 shows the average [mean (SD)] and median [min, max] number of days on which exclusive human milk feeds were dispensed. Infants had a median hospital stay of 47 days, with a median of 56 days until the end of the BPD observation period. For infants born at <28 weeks GA, both the average duration of hospitalization and the time to the end of the BPD period were longer compared to those born between 28 and 32 weeks (Supplementary Table S1). All infants had at least 1 day on which exclusive human milk feeds were dispensed. For instance, exclusive MOM was dispensed on at least 1 day for *n* = 758 infants, on an average of 34% of the days of hospitalization until BPD classification (Table 2). Exclusive fresh milk feeds were dispensed for at least 1 day to *n* = 788 infants covering a median of 37% of hospitalization days during the observation period. On average, exclusive fresh raw human milk was dispensed on 22% of the days.

**Table 2:** Days on which exclusive feeds were dispensed during hospitalization in the observational period.

	Infants (*n*)	Mean (SD)	Median [min, max]
Number of days of the whole hospitalization	866	54 (33)	47 [2, 430]
Number of days of hospitalization in the observational period until classification of BPD	866	51 (14)	56 [29, 91]
% of days of hospitalization until classification of BPD with…			
Exclusive human milk feeds	866	74% (27%)	89% [1, 100%]
Other nutrition	850	26% (27%)	12% [1, 99%]
Fresh milk*	788	40% (26%)	37% [1, 95%]
Fresh raw milk	632	22% (19%)	16% [1, 84%]
Fresh raw MOM	580	17% (19%)	17% [1, 84%]
Fresh raw DHM	118	5% (5%)	3% [1, 25%]
MOM	758	34% (30%)	47% [1, 96%]
DHM	793	20% (22%)	11% [1, 99%]
MOM and DHM	563	20% (21%)	10% [1, 88%]

BPD, Bronchopulmonary dysplasia; DHM, Donor human milk; GA, Gestational age; MOM, Mothers’ own milk. ^*^Fresh milk, Milk is stored cold for a maximum of 3 days, both MOM and DHM included. Fresh raw milk refers to fresh unpasteurized MOM or DHM.

**Figure 2:**
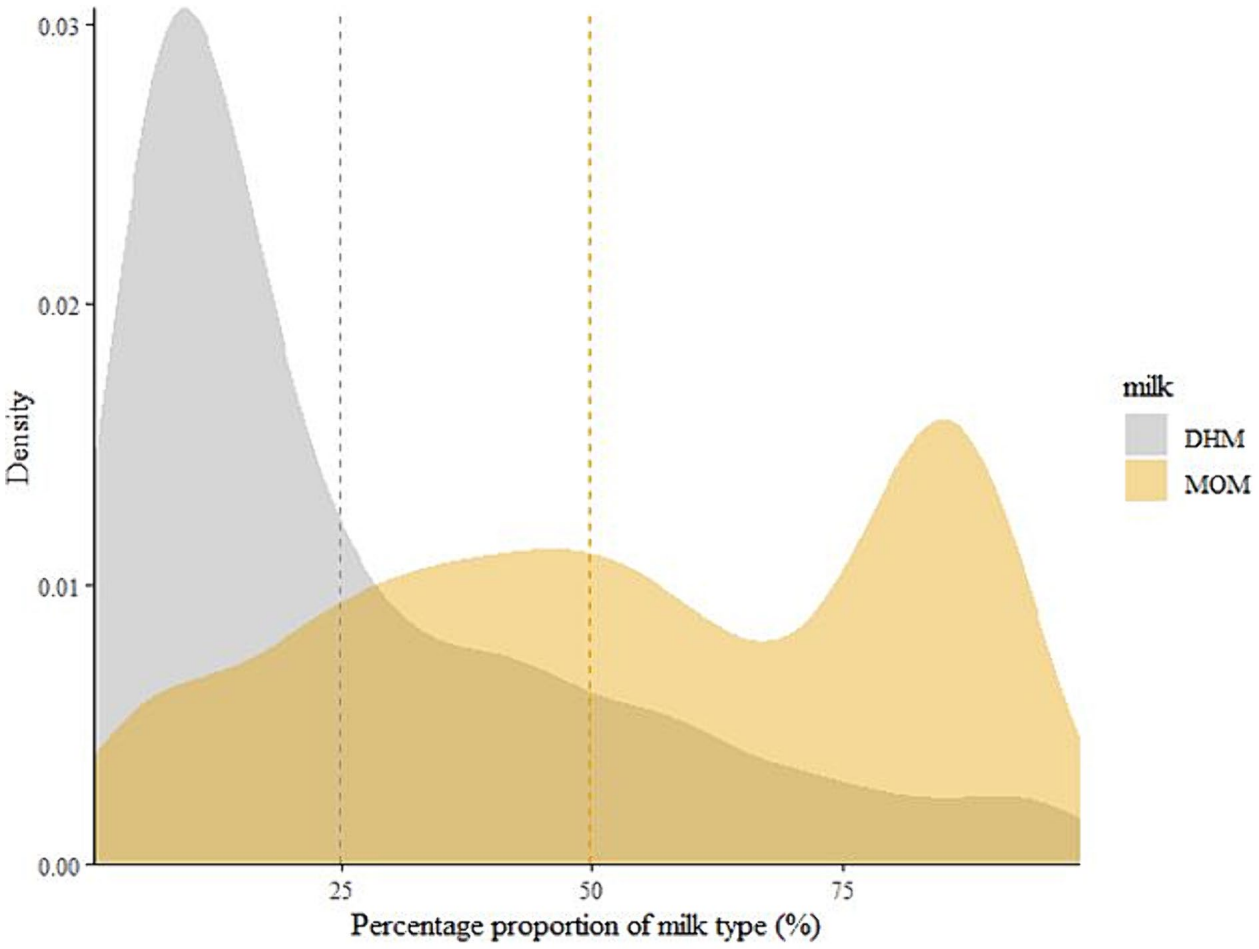
The distribution of mother’s own milk (MOM) and donor human milk (DHM) contributions to the overall dispensed milk feeds for an infant during hospitalization until the end of the observational period to classify BPD. Dotted lines represent the means of the contribution of the respective human milk feed as a percentage of the days on which any human milk was dispensed. DHM, Donor human milk; MOM, Mothers’ own milk.

Density plots were created to visualize the distribution of the dispensed human milk feeds (Figure 2; Supplementary Figures S1–S3). DHM contributed less to overall dispensed milk feeds compared to MOM. More than 75% DHM was dispensed for only a few infants, while most infants had less than 50% DHM dispensed. Conversely, the average proportion of MOM was balanced and slightly shifted toward higher percentages (>50%) with a peak well above 75% MOM.

Infants were categorized into one of five human milk categories: fresh vs. frozen milk, pasteurized vs. raw milk, MOM vs. DHM, and raw MOM vs. raw DHM. Table 3 shows the percentage contributions of each milk type within in each milk group to which the infants were assigned. Infants belonging to the fresh milk group had a higher fraction of MOM compared to the frozen group, while the frozen group had a larger DHM proportion. Percentage contributions of pasteurized and raw milk also differed between these subgroups. Within the pasteurized milk group, the average MOM proportion and the proportion of fresh milk were lower compared to the raw milk group. In the MOM group, the proportion of fresh milk and raw milk was greater than in the DHM group.

**Table 3:** Proportions of dispensed milk feeds within the different human milk groups used in the current analysis.

Group	Proportion of milk type	Average (%)	% Median [Q1–Q3]
Fresh milk	MOM	78	82 [68–88]
	DHM	15	11 [7–19]
	Pasteurized Milk	42	41 [20–65]
	Raw Milk	50	52 [29–72]
Frozen milk	MOM	32	32 [18–44]
	DHM	65	63 [53–78]
	Pasteurized Milk	54	54 [42–68]
	Raw Milk	36	35 [21–49]
Pasteurized milk	MOM	58	58 [38–81]
	DHM	40	37 [15–58]
	Fresh milk	52	53 [34–74]
	Frozen milk	41	38 [20–58]
Raw milk	MOM	73	82 [65–88]
	DHM	21	11 [8–21]
	Fresh milk	62	69 [50–78]
	Frozen milk	30	23 [16–38]
MOM	Pasteurized Milk	40	39 [18–62]
	Raw Milk	50	51 [31–70]
	Fresh milk	67	70 [56–79]
	Frozen milk	23	21 [13–30]
DHM	Pasteurized Milk	55	59 [43–70]
	Raw Milk	34	31 [19–47]
	Fresh milk	22	22 [9–35]
	Frozen milk	69	67 [57–79]
Raw MOM	Fresh milk	59	61 [43–77]
	Frozen milk	21	18 [11–27]
Raw DHM	Fresh milk	29	27 [12–46]
	Frozen milk	59	58 [42–75]

Infants were categorized into one of five human milk categories: fresh vs. frozen milk, pasteurized vs. raw milk and MOM vs. DHM, and raw MOM vs. raw DHM. Percentages shown refer to the contribution of each milk type to the respective categories in bold. MOM, Mother’s own milk; DHM, Donor human milk.

**Figure 3:**
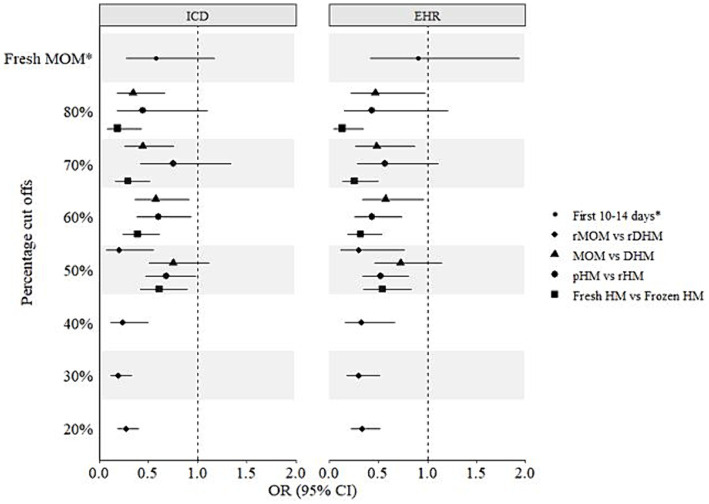
Crude associations of different milk types and bronchopulmonary dysplasia (BPD) in preterm infants. ICD, International Statistical Classification of Diseases and Related Health Problems; EHR, Electronic hospital records; CI, Confidence interval; DHM, Donor human milk; HM, Human milk; MOM, Mothers’ own milk; OR, Odds ratio; pHM, Pasteurized human milk; rDHM, Raw donor human milk; rHM, Raw human milk; rMOM, Raw mothers’ own milk; *Fresh MOM in the first 10–14 days vs. any DHM in the first 10–14 days.

**Figure 4:**
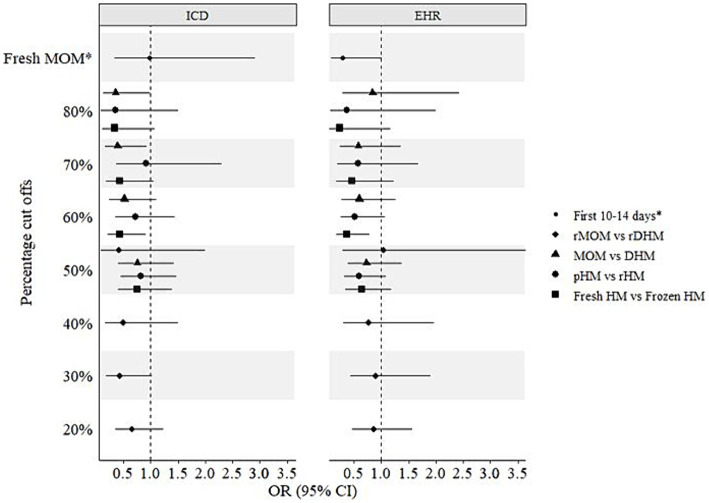
Adjusted associations of different milk types and bronchopulmonary dysplasia (BPD) in preterm infants. ICD, International Statistical Classification of Diseases and Related Health Problems; EHR, Electronic hospital records; CI, Confidence interval; DHM, Donor human milk; HM, Human milk; MOM, Mothers’ own milk; OR, Odds ratio; pHM, Pasteurized human milk; rDHM, Raw donor human milk; rHM, Raw human milk; rMOM, Raw mothers’ own milk; *Fresh MOM in the first 10–14 days vs. any DHM in the first 10–14 days.

In addition to the above-mentioned four human milk groups, infants were further grouped into cut offs ranging between 20 and 80% of the respective milk feeds. The fifth group was a comparison between fresh MOM vs. any DHM in the first 14 days (Figure 3). In crude models, statistically significant (*p* < 0.05) protective associations of 50–80% fresh vs. frozen milk, 60–80% MOM vs. DHM, and 20–50% raw MOM vs. raw DHM were observed using both BPD operationalizations (Figure 3). Interestingly, 50–60% pasteurized milk showed lower odds of developing BPD (*p* < 0.05). However, following adjustment for gestational age, birth weight, child sex, surfactant therapy, and pre- and postnatal steroid therapy, most associations were not statistically significant (*p* > 0.05) except for 70–80% MOM vs. DHM, 60% fresh vs. frozen milk using the ICD- and EHR-based BPD operationalizations, respectively (Figure 4). In infants with a GA <28 weeks, 60% fresh milk vs. frozen milk was associated with lower odds of BPD using the EHR-based definition, in both crude and adjusted models (Supplementary Figures S4, S5).

## 4 Discussion

The current study investigated associations between dispensed human milk feeds and the odds of BPD in preterm infants, considering different milk types and percentage cut-off values, along with two BPD operationalizations. Results showed a significant protective association of fresh human milk compared to frozen, as well as MOM compared to DHM. Interestingly, pasteurized milk showed a protective association against BPD compared to raw milk, although not statistically significant.

We found lower odds of BPD with fresh compared to frozen milk across different percentage cutoffs, particularly notable with higher proportions of fresh milk. Notably, the fresh milk group contained more MOM and raw milk, while the frozen milk group had more DHM and pasteurized milk. Thereby potentially influencing these associations. With a similar limitation, a randomized controlled trial (RCT) comparing frozen MOM to both fresh and frozen MOM and found no difference in BPD rates, though BPD was not the primary focus (24). Similarly, Sun et al. (33) observed faster weight gain, lower incidence of retinopathy of prematurity (ROP), and reduced BPD in the fresh milk group, suggesting potential benefits of fresh human milk for improving infant outcomes.

Conversely, our study shows a protective association of pasteurized human milk, although not statistically significant, following adjustment for confounding factors. A systematic review (34), found no BPD difference between unpasteurized and pasteurized human milk, while a recent prospective study (35) showed that preterm infants fed fresh MOM compared to pasteurized, non-frozen MOM, had a higher survival rate without severe complications, including BPD. In contrast to our study, that study (35) implemented a MOM-only nutrition regime (supplemented with preterm formula if necessary). Similarly, two other studies (20, 21) found a reduced risk of BPD in the raw MOM group compared to the pasteurized MOM group. In our study, the proportion of MOM and fresh milk dispensed among pasteurized milk were more than 50%, although not higher than those proportions among dispensed raw milk. It is therefore plausible that our results were confounded by the proportion of dispensed MOM and fresh milk.

Moreover, in contrast to previous studies that showed inconclusive results and no beneficial effects of MOM (19, 31, 36–38), we report a protective association of MOM vs. DHM. However, it must be noted that BPD was only considered as a secondary outcome in most of these studies (36–38), which comes with different observational periods compared to our study design. On the other hand, one study (19) stood out for its detailed information on actual human milk intake during the first 28 days of life, as well as proportion of MOM on total breast milk intake. But these results, similarly to ours, should be interpreted with caution as the sample size was very small and only few infants received DHM.

Xu et al. (31) demonstrated a protective effect of >50 mL/kg/day of human milk, both MOM and DHM, against BPD compared to preterm formula. Similarly, another study (39), found a 9.5% reduction in the odds of BPD for every 10% increase in MOM dose. Fonseca et al. (40) reported a protective effect of 7 mL/kg/day of human milk in the first 42 days of life regarding BPD. However, all human milk in that study (40) was frozen and pasteurized. Although we lacked actual volume data and infant intake, we reported a protective association of fresh MOM dispensed in the first 10–14 days of life against BPD. While not statistically significant, this highlights the importance of early exposure to MOM in the first few days of life.

This study has several limitations that should be acknowledged. Firstly, the retrospective nature and observational design of the study inherently limit the ability to establish causality. Secondly, the data on dispensed human milk feeds, does not accurately reflect actual infant feeding practices and there is no information on the volume of actual human milk intake. Thus, a potential dose dependent association could not be investigated. Still, we accounted for the potential changes in feeding type by applying weighting to the data on dispensed human milk feeds. This resulted in plausible intra-individual feeding patterns during the first 100 days of hospitalization (32). Additionally, due to small numbers of infants with BPD in the gestational age categories, our stratified analysis lacked sufficient power, limiting the possibility to draw robust conclusions. Moreover, most BPD cases were mild (*n* = 174), with even fewer moderate (*n* = 17) and severe BPD (*n* = 19), making it challenging to assess BPD severity accurately. While we did evaluate severity, (Supplementary Figures S6, S7), caution when interpreting these results is warranted due to limited sample sizes. Further, there is no data on calorie and/or protein intake and growth parameters in the study population. Of which, protein intake and growth could have a positive impact on BPD. Despite this, we provide valuable insights and highlight the need for further prospective studies that can incorporate comprehensive data on nutritional intake and growth parameters to better understand their impact on BPD. Our study’s strength includes the determination of the relative contribution of each milk type to the respective group, providing clearer insights of the feed mix compared to previous research. This tackles a key issue of merging MOM and DHM in single matrices, reducing inclusion bias and enhancing generalizability in observational analyses. We also employed various cutoff values tailored to our population and clinical setting (32). Additionally, we used two distinct operationalizations of BPD aligning with previous studies (one stricter based on NICHD consensus and one more relaxed based on administrative data), which yielded similar results. However, residual confounding from unaccounted factors (as they were not available) may influence results, although we believe their impact to be minimal.

In conclusion, our findings suggest potential protective associations of higher contribution of fresh human milk and MOM to overall feeds during hospitalization against developing BPD. Our results highlight the importance of MOM as an ideal source of nutrition during early infancy. The dose dependent effect of these associations still remains to be studied. For future research, data on the actual amount of milk feeds should be analyzed to verify these findings. Future research could examine whether the initiation time and duration of MOM feedings has an impact on short-term outcomes including BPD.

## Data availability statement

The data analyzed in this study are subject to the following licenses/restrictions: due to data protection laws, we may not be able to share the raw data. However, the authors are open to sharing aggregate data (for instance, relative concentrations of the different milk types). Requests to access these datasets should be directed to jon.genuneit@medizin.uni-leipzig.de.

## Ethics statement

The studies involving humans were approved by the Ethics Board of the Medical Faculty at Leipzig University. The studies were conducted in accordance with the local legislation and institutional requirements. The ethics committee/institutional review board waived the requirement of written informed consent for participation from the participants or the participants’ legal guardians/next of kin because written informed consent for participation was not required from the participants or the participants’ legal guardians/next of kin in accordance with the national legislation and institutional requirements.

## Author contributions

EP: Conceptualization, Data curation, Formal analysis, Investigation, Methodology, Writing – original draft, Writing – review & editing. RA: Data curation, Investigation, Methodology, Writing – review & editing. TW: Data curation, Investigation, Methodology, Writing – review & editing. UT: Data curation, Investigation, Methodology, Writing – review & editing. CG: Conceptualization, Data curation, Formal analysis, Investigation, Methodology, Writing – review & editing. JG: Conceptualization, Data curation, Formal analysis, Investigation, Methodology, Writing – original draft, Writing – review & editing. LS: Conceptualization, Data curation, Formal Analysis, Investigation, Methodology, Project administration, Writing – original draft, Writing – review & editing.
