# Supplementary material for: The association of different types of human milk with bronchopulmonary dysplasia in preterm infants
Source: Front Nutr. 2024 Aug 7;11:1408033. doi: 10.3389/fnut.2024.1408033 (PMC11337300; doi:10.3389/fnut.2024.1408033)
Supplement: Supplementary file 1 [file Data_Sheet_1.docx]

Supplementary Material

**The association of different types of human milk with bronchopulmonary dysplasia in preterm infants**

Elisabeth Pütz^1^, Rudolf Ascherl^2^, Thomas Wendt^3^, Ulrich H. Thome^2^, Corinna Gebauer^2^, Jon Genuneit^1,4^, Linda P. Siziba^1^

^1^Pediatric Epidemiology, Department of Pediatrics, Medical Faculty, Leipzig University, Leipzig, Germany

^2^Division of Neonatology, Department of Pediatrics, University of Leipzig Medical Centre, Leipzig, Germany

^3^Data Integration Centre, University of Leipzig Medical Centre, Leipzig, Germany

^4^German Center for Child and Youth Health (DZKJ)

**Table S1**: Days on which exclusive feeds were ordered during hospitalization until bronchopulmonary dysplasia (BPD) period end stratified by gestational age

|  | GA < 28 weeks | | | GA ≥28 and ≤ 32 weeks | | |
| --- | --- | --- | --- | --- | --- | --- |
|  | **Infants (n)** | **Mean (sd)** | **Median**  **[min, max]** | **Infants (n)** | **Mean (sd)** | **Median**  **[min, max]** |
| Number of days of whole hospitalization | 226 | 83 (30) | 82 [5, 200] | 640 | 43 (27) | 40 [2, 430] |
| Number of days of hospitalization in the observational period to classify BPD | 226 | 70 (8) | 69 [57, 91] | 640 | 45 (10) | 46 [29, 56] |
| % of days of hospitalization during the observational period for classification of BPD with… |  |  |  |  |  |  |
| exclusive human milk feeds | 226 | 82% (23%) | 93% [1%, 100%] | 640 | 71% (27%) | 83% [2%, 100%] |
| other nutrition | 221 | 18% (23%) | 7% [1%, 99%] | 629 | 29% (27%) | 19% [1%, 98%] |
| fresh milk* | 210 | 41% (26%) | 41% [1%, 93%] | 578 | 40% (26%) | 35% [2%, 95%] |
| fresh raw milk | 169 | 22% (20%) | 16% [1%, 82%] | 463 | 21% (19%) | 16% [1%, 84%] |
| fresh raw MOM | 145 | 17% (20%) | 19% [1%, 82%] | 435 | 17% (19%) | 17% [1%, 84%] |
| fresh raw DHM | 51 | 5% (6%) | 2% [1%, 25%] | 67 | 4% (3%) | 3% [2%, 20%] |
| MOM | 202 | 39% (31%) | 59% [1%, 96%] | 556 | 34% (29%) | 46% [2%, 96%] |
| DHM | 206 | 26% (28%) | 12% [1%, 99%] | 587 | 17% (20%) | 10% [2%, 98%] |
| MOM and DHM | 128 | 17% (21%) | 7% [1%, 88%] | 435 | 20% (21%) | 11% [1%, 84%] |

BPD: Bronchopulmonary dysplasia; DHM: Donor human milk; GA: Gestational age; MOM: Mothers' own milk. * Fresh milk = Milk is stored cold for a maximum of 3 days, both MOM and DHM included.


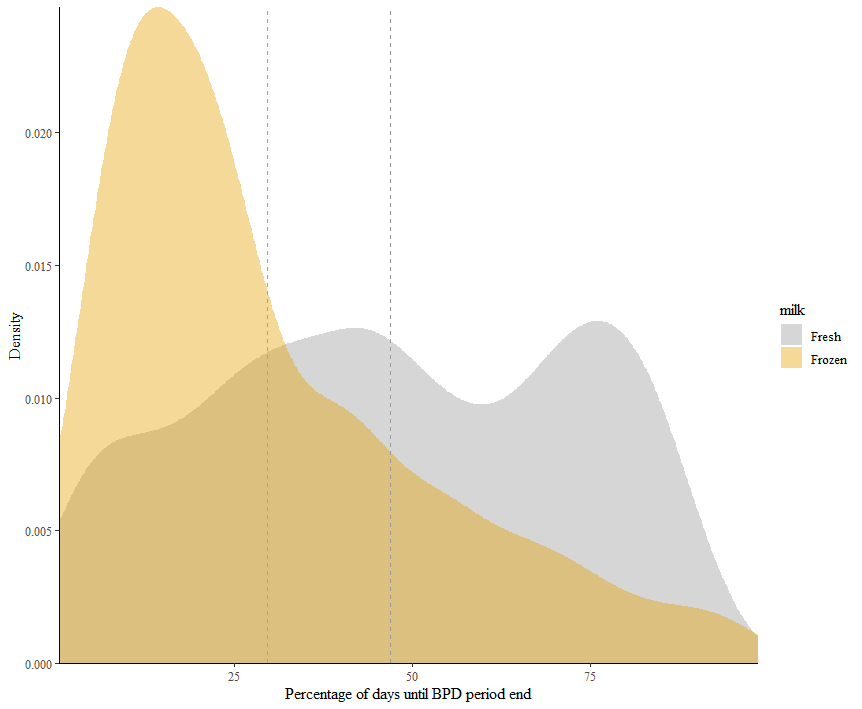


**Figure S1:** The distribution of fresh milk and frozen milk contributions to the overall dispensed milk feeds for an infant during hospitalization in the observational period to classify BPD. Dotted lines represent the means of the contribution of the respective human milk feed as a percentage of the days on which any human milk was ordered. BPD: Bronchopulmonary Dysplasia


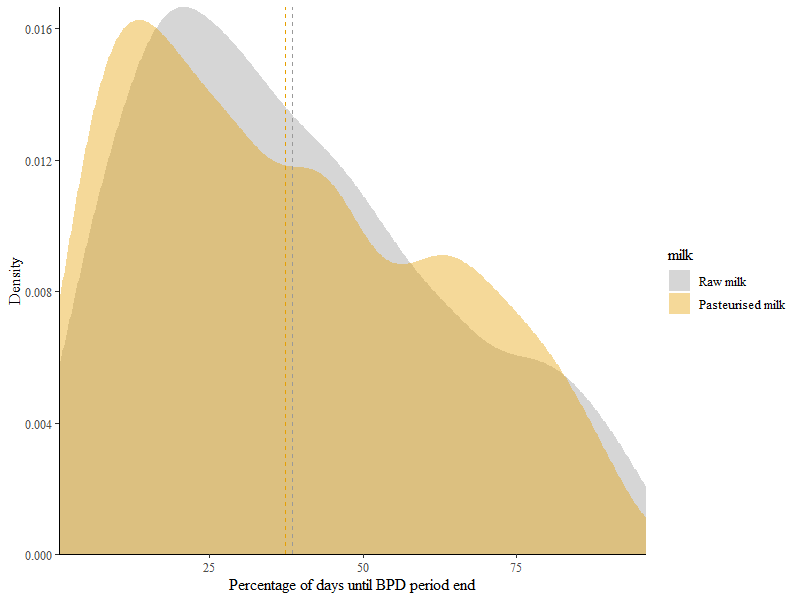


**Figure S2:** The distribution of raw milk and pasteurised milk contributions to the overall ordered milk feeds for an infant during hospitalization in the observational period to classify BPD. Dotted lines represent the means of the contribution of the respective human milk feed as a percentage of the days on which any human milk was ordered. BPD: Bronchopulmonary Dysplasia


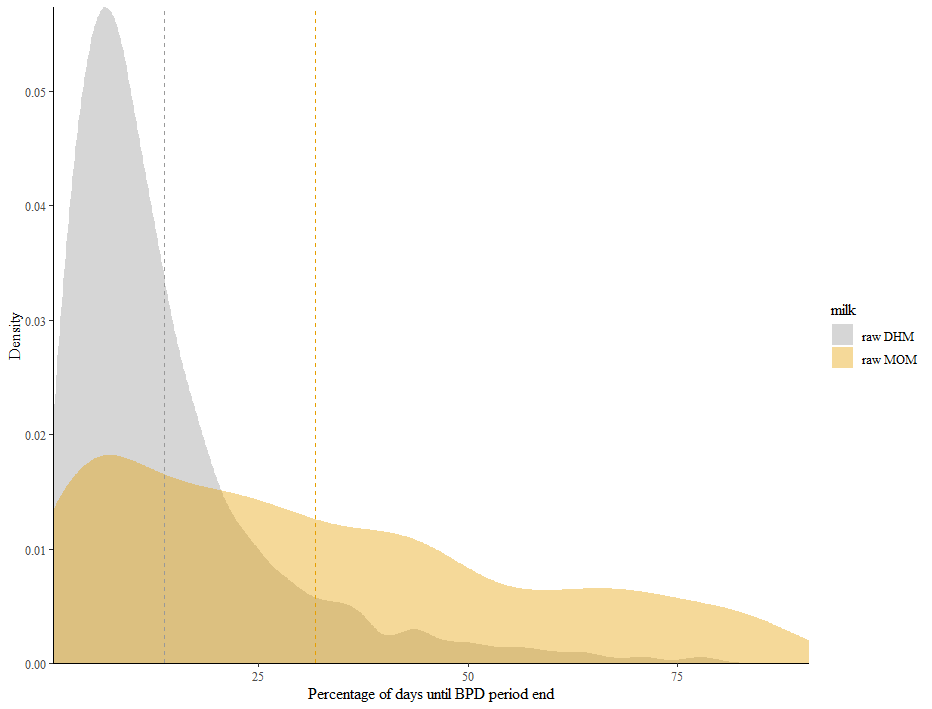


**Figure S3:** The distribution of raw mother’s own milk (MOM) and raw donor human milk (DHM) contributions to the overall ordered milk feeds for an infant during hospitalization in the observational period to classify BPD. Dotted lines represent the means of the contribution of the respective human milk feed as a percentage of the days on which any human milk was ordered. DHM: donor human milk, MOM: mothers' own milk


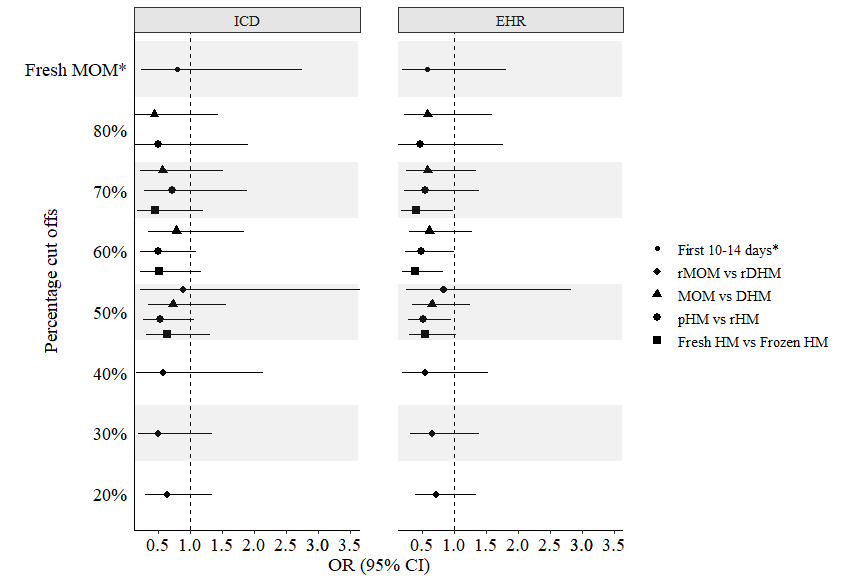


**Figure S4:** Crude associations of different milk types and bronchopulmonary dysplasia (BPD) in preterm infants born at gestational age less than 28 weeks**.** CI: confidence interval; DHM: donor human milk; HM: human milk; MOM: mothers' own milk; OR: odds ratio; pHM: pasteurized human milk; rDHM: raw donor human milk; rHM: raw human milk; rMOM: raw mothers' own milk; ICD: International Statistical Classification of Diseases and Related Health Problems. EHR: Electronic hospital records. * Fresh MOM vs any DHM in the first 10-14 days


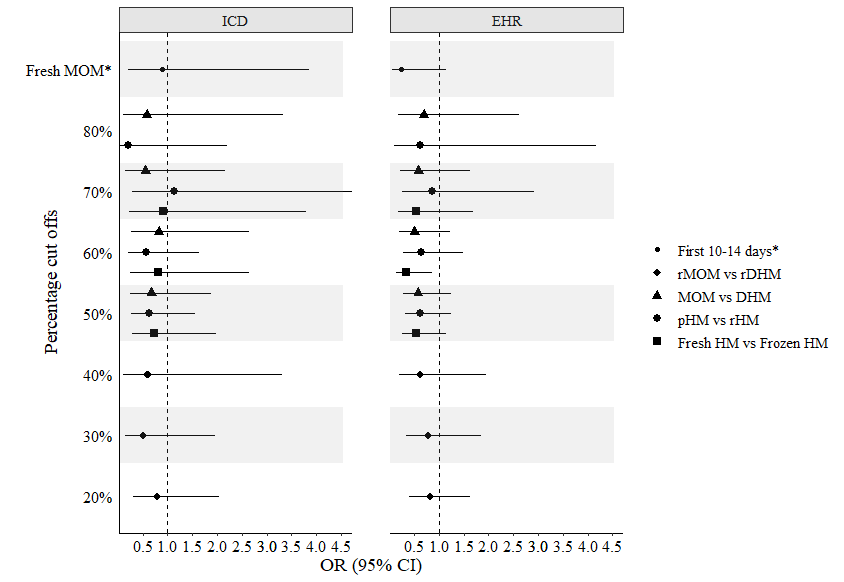


**Figure S5:** Adjusted associations of different milk types and bronchopulmonary dysplasia (BPD) in preterm infants born at gestational age less than 28 weeks**.** CI: confidence interval; DHM: donor human milk; HM: human milk; MOM: mothers' own milk; OR: odds ratio; pHM: pasteurized human milk; rDHM: raw donor human milk; rHM: raw human milk; rMOM: raw mothers' own milk; ICD: International Statistical Classification of Diseases and Related Health Problems.; EHR: Electronic hospital records. * Fresh MOM vs any DHM in the first 10-14 days

**Figure S6**: Crude associations of different milk types and bronchopulmonary dysplasia (BPD) severity in preterm infants**.** ICD: International Statistical Classification of Diseases and Related Health Problems; EHR: Electronic hospital records; CI: confidence interval; DHM: donor human milk; HM: human milk; MOM: mothers' own milk; OR: odds ratio; pHM: pasteurized human milk; rDHM: raw donor human milk; rHM: raw human milk; rMOM: raw mothers' own milk; * Fresh MOM in the first 10-14 days vs any DHM in the first 10-14 days

**Figure S7:** Adjusted associations of different milk types and BPD in preterm infants**.** ICD: International Statistical Classification of Diseases and Related Health Problems; EHR: Electronic hospital records; CI: confidence interval; DHM: donor human milk; HM: human milk; MOM: mothers' own milk; OR: odds ratio; pHM: pasteurized human milk; rDHM: raw donor human milk; rHM: raw human milk; rMOM: raw mothers' own milk; * Fresh MOM in the first 10-14 days vs any DHM in the first 10-14 days.
